# Analysis of the Motion Characteristics of Coarse Aggregate Simulated by Smart Aggregate During the Compaction Process

**DOI:** 10.3390/ma18051143

**Published:** 2025-03-04

**Authors:** Xiaofeng Wang, Feng Wang, Xiang Li, Shenghao Guo, Yi Zhou

**Affiliations:** 1 School of Materials Science and Engineering, Wuhan University of Technology, Wuhan 430070, China; wxf@whut.edu.cn (X.W.); 330994@whut.edu.cn (X.L.); joejoe-98@whut.edu.cn (Y.Z.); 2 School of Materials Science and Engineering, Chang’an University, Xi’an 710064, China; gshenghao@chd.edu.cn

**Keywords:** asphalt pavement, degree of compaction, 3D printing, intelligent aggregates, kinematic characteristics of coarse aggregates

## Abstract

Asphalt pavement has become a vital component of modern highway construction due to its high wear resistance, short construction period, economic viability, and excellent skid resistance. However, increasing traffic volume has heightened the structural performance requirements of asphalt pavement, especially during compaction. The compaction degree of asphalt mixtures has emerged as a key indicator for assessing construction quality. This study explores the relationship between the internal structural evolution of asphalt mixtures and their compaction performance, focusing on the motion behavior of coarse aggregates. To achieve this, a wireless smart aggregate was developed using 3D printing technology to simulate coarse aggregate motion and enable real-time monitoring during compaction. Compaction experiments, including Superpave gyratory compaction and wheel rolling, were conducted on asphalt mixtures with different gradations (e.g., AC-13 and AC-20). The dynamic responses of smart aggregates were analyzed to identify motion patterns. The results show that the Superpave gyratory compaction method more accurately replicates aggregate motion observed in road construction. Additionally, asphalt mixture gradation significantly affects the motion behavior of coarse aggregates. This study provides insights into the microscale motion of coarse aggregates and its connection to compaction performance, contributing to improved asphalt pavement quality and efficiency.

## 1. Introduction

Asphalt pavement, one of the primary forms of modern highway construction, offers several advantages, including high wear resistance, a short construction period, economic feasibility, excellent anti-skid performance, and low noise levels [1]. With the rapid development of the global economy, transportation volumes continue to rise, leading to increasingly higher demands for asphalt pavement structures. Compaction, a crucial process that impacts the structural strength of asphalt pavement, involves applying external pressure to transform loose asphalt mixtures into a dense state. In the compaction process, the degree of compaction of the asphalt mixture is a vital indicator for assessing the quality of asphalt pavement construction [2]. Appropriate compaction conditions can enhance the structural strength and durability of asphalt pavement, improve its resistance to rutting, and minimize permanent deformation under vehicle loads. Consequently, investigating the relationship between the evolution of the internal structure of asphalt mixtures and their compaction performance—particularly the stress conditions of coarse aggregates at the microscale—can significantly enhance the compaction quality of asphalt pavements.

Currently, during the construction of asphalt pavement, technicians typically rely on their personal experience or construction techniques suggested by relevant industry standards to manage compaction quality. However, there is often a lack of understanding regarding the movement dynamics and mechanisms of the aggregates within the asphalt mixture during the compaction process. As a result, they are unable to design asphalt pavements that are tailored to the specific needs and conditions of local construction environments. The inspection of compaction quality is typically conducted after the compaction process, with the degree of compaction considered a key indicator of pavement quality. Common methods used include core sampling and nuclear density meters [3], with some employing ground-penetrating radar to assess the internal structure of the pavement [4,5]. While these techniques can provide insights into the internal structure of compacted pavement, they may also compromise the overall integrity of the pavement and do not allow for precise control over the rolling frequency during asphalt pavement construction. Moreover, equipment like ground-penetrating radar remains impractical for assessing the compaction degree in the asphalt pavement compaction process due to its high cost and cumbersome operation.

To achieve more in-depth monitoring of the internal conditions of the road surface, researchers have further developed technologies such as fiber optic sensors, piezoelectric sensors, and SmartRock sensors. Tan et al. [6] demonstrated the potential of FBG fiber Bragg grating sensors for road surface monitoring through real-time monitoring of the pavement of the Wusu Bridge. Monsberger et al. [7] tested distributed fiber optic shape sensors embedded in concrete beams and full-scale tunnel lining structures. Their findings confirmed that these sensors can accurately monitor shape deformation before significant cracks develop in the structures, thereby facilitating a model-free evaluation of the entire distributed cross-section. Liu et al. [8] used FBG sensing technology to embed fiber Bragg gratings into the pavement structure layer, revealing the variation law of dynamic strain response of asphalt pavement. The experimental results showed the rationality and feasibility of evaluating the actual mechanical response of pavement structure through on-site testing of fiber Bragg grating sensors. Shen et al. [9] used fiber Bragg grating sensors to analyze the mechanical response of semi-rigid base asphalt pavement, flexible base asphalt pavement, and composite base asphalt pavement structures. The results indicate that fiber Bragg grating sensors can be effectively applied in different types of road structures. Zhang et al. [10] improved the distributed fiber Bragg grating sensor and proposed a flexible configuration of distributed fiber shape sensors for accurate health monitoring of road structures. Zhang et al. [11] embedded piezoelectric ceramic sensors inside concrete for long-term monitoring, and experimental data showed that the prepared piezoelectric ceramic sensors can effectively monitor changes in the internal structure of concrete. Sun et al. [12] used piezoelectric ceramic sensors bonded to concrete beams for structural health monitoring and studied the effects of uniaxial compressive stress and the resulting internal cracking of concrete on the waveform amplitude received by the piezoelectric ceramic sensors. The experimental structure showed that piezoelectric ceramic sensors have the potential to monitor cracking and long-term deterioration of concrete structures. Wang et al. [13,14] used embedded SmartRock sensors to monitor particle movement during the compaction process of asphalt mixtures in laboratories and construction sites. As a result, it was found that SmartRock sensors can achieve wireless data transmission and obtain motion information inside the road surface, and their small size has less impact on the road structure. Dan et al. [15] also analyzed the aggregate movement characteristics inside asphalt concrete by embedding SmartRock sensors into asphalt mixtures and conducting Superpave gyratory compaction experiments. The experimental data showed that there is a significant linear correlation between the data collected by SmartRock sensors and the compaction degree of asphalt mixtures after analysis. Huang et al. [16] independently designed and developed an intelligent aggregate with a regular tetrahedral shape based on the actual usage needs of intelligent aggregates for asphalt pavement. Zhang et al. [17,18] conducted tests on asphalt pavement construction sites by introducing intelligent aggregates, and the results showed that the microscopic response of the tests not only reflected the compaction status on-site, but also timely judged whether there was any leakage throughout construction.

**Figure 1:**
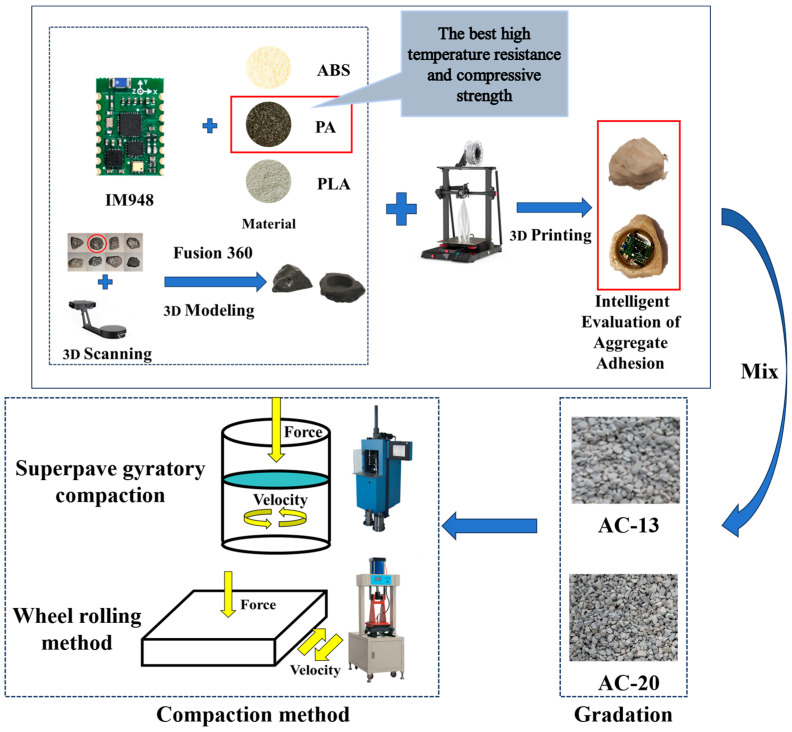
Technology road map.

In summary, although various sensors have been applied in road surfaces to a certain extent, due to their usually rectangular shape, they cannot simulate the motion characteristics of coarse aggregates during the compaction process of asphalt mixtures, and due to incomplete technology, they cannot be mass-produced and promoted. Therefore, after consulting the relevant literature [13,14], it was decided to use 3D printing technology for the manufacturing of intelligent aggregate shells, and the selection of shell shape was completed through 3D scanning and Fusion 360 processing. This study aims to develop a self-made wireless intelligent aggregate. This type of intelligent aggregate mainly consists of three parts: attitude sensor, power supply, and packaging shell. The packaging shell of intelligent aggregates is made by 3D scanning the shape of natural aggregates and using 3D printing technology. This intelligent aggregate is mainly compressed by the contact between the aggregate and the sensor housing during the compaction process, which causes the position of the intelligent aggregate to change, thereby obtaining sensor attitude data to further analyze and study the motion characteristics of coarse aggregate during the compaction process. So, it can simulate the dynamic response of coarse aggregates at the time of the compaction process of asphalt concrete pavement, thereby monitoring the pavement in real time and assisting in ensuring the construction quality of the pavement. The technical road map of this study is shown in Figure 1. This experimental study is expected to provide some new clues for the intrinsic relationship between the movement characteristics of coarse aggregates and the compaction performance of asphalt mixtures at the microscale.

## 2. Materials and Methods

### 2.1. Raw Materials

#### 2.1.1. Intelligent Aggregates

**Figure 2:**
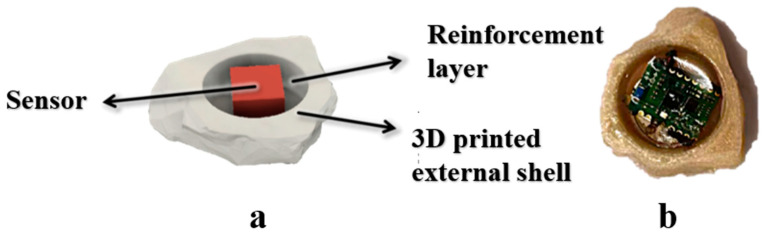
Intelligent aggregate: (**a**) intelligent aggregate model; (**b**) actual intelligent aggregate.

SmartRock was initially applied in railway engineering to detect the dynamic response of foundations under train loads. However, due to its high cost and large volume, it is not well-suited for simulating the motion characteristics of coarse aggregates during compaction, particularly because of its cubic shape [19] Therefore, this article presents a custom-designed intelligent aggregate that closely resembles real coarse aggregate particles. This innovative aggregate is capable of forming the skeletal structure of the road surface in conjunction with an asphalt mixture after rolling. The intelligent aggregate is primarily composed of three components: sensors, reinforcement layers, and a 3D printed external shell, as shown in Figure 2.

**Figure 3:**
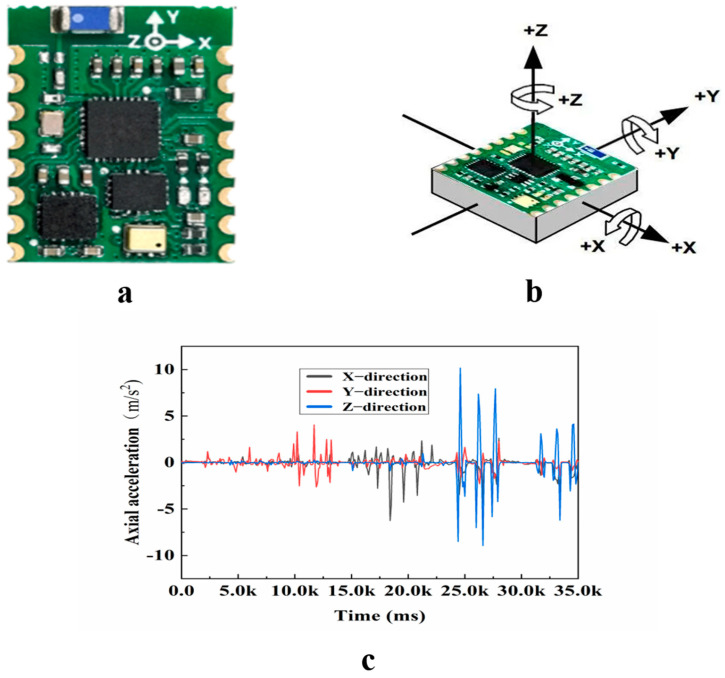
IM600 sensor (Shenzhen, China): (**a**) sensor physical image; (**b**) sensor coordinate system; (**c**) sensor PC data acquisition.

Intelligent aggregates serve as a component of pavement structures in practical engineering; therefore, the selection of these aggregates must satisfy specific criteria. Over the course of the mixing process, the temperature of the asphalt typically ranges from 140 to 160 °C. Therefore, when selecting sensors and batteries, high temperature-resistant batteries and high-performance six-axis acceleration sensors are chosen (Figure 3). To analyze the motion of coarse aggregate during the compaction process of an asphalt mixture, sensors are utilized to measure parameters such as angle, angular velocity, and changes in acceleration under load. A six-axis accelerometer consists of a three-axis accelerometer and a three-axis gyroscope. In addition to measuring an object’s acceleration across three axes, it can also detect changes in the object’s direction and angle [20]. Furthermore, this sensor is compact and lightweight, which effectively reduces the volume of smart aggregates, allowing them to better match the size of natural aggregates. (The sensor is the IM948, which was purchased from the technology industry). The sensor parameters are presented in Table 1.

The reinforcement layer and packaging shell of intelligent aggregates must possess sufficient compressive strength, high temperature resistance, corrosion resistance, high stability, and ease of processing. This study employs a high temperature-resistant epoxy resin with excellent mechanical properties for the reinforcement layer. The primary parameters of this resin are summarized in Table 2. The protective shell is fabricated using 3D printing technology, necessitating the selection of appropriate 3D printing materials.

ABS plastic is a ternary copolymer composed of three monomers: acrylonitrile (A), butadiene (B), and styrene (C). It exhibits the combined properties of these components, including chemical corrosion resistance, heat resistance, and high elasticity. Additionally, ABS plastic possesses a degree of surface hardness and has a processing temperature range of approximately 210–240 °C. However, it demonstrates relatively low bending and compressive strength. The main parameters are summarized in Table 3. Currently, ABS plastic is primarily utilized in the fields of automobiles, electronic appliances, and building materials [21]. In the context of highway engineering, Wang [13,14] employed ABS plastic as the outer packaging material for SmartRock, yielding promising results.

Polylactic acid (PLA) is a novel biodegradable material characterized by good thermal stability and flame retardancy. It has a processing temperature range of 170–230 °C and exhibits strong solvent resistance, remaining insoluble in asphalt. The main parameters of PLA are summarized in Table 4. It is commonly used for crafting handicrafts, art pieces, and experimental models. Its physical, thermal, and mechanical properties are closely linked to its molecular distribution, molecular weight, and stereochemistry, which allow it to exist in either an amorphous or a semi-crystalline state. By employing controlled polymerization of various optical monomers, different types of PLA can be synthesized, each exhibiting unique properties. To overcome the inherent hardness limitations of PLA, researchers are exploring blends with 3D composite materials such as PLLA/PDLA to enhance its mechanical and thermal properties [22].

Polyamide (PA), commonly known as nylon, is the world’s first synthetic fiber. It boasts a range of excellent properties, including excellent mechanical property, heat resistance, wear resistance, and chemical stability. Nylon also exhibits a degree of flame retardancy and is easy to process, with a typical processing temperature of approximately 240–260 °C. Its main technical parameters are outlined in Table 5. Consequently, nylon is well suited for a variety of applications, including industrial components, gears, tents, and parts such as aircraft wings and engine hoods. Its versatility has made nylon an indispensable material in many industries and a vital element in modern production and daily life [23,24].

According to the ASTM Standards (ASTM D6373) [25], the ambient temperature of the asphalt mixture during hot mix asphalt pavement paving can reach between 140 °C and 160 °C. Therefore, the high temperature stability of the packaging material for intelligent aggregate is particularly crucial. In comparing the thermal deformation temperatures and printing temperatures of three different printing materials, this study ultimately selected polyamide, which exhibits the highest thermal deformation temperature and has a printing temperature range of 240 °C to 260 °C, as the preferred packaging material for smart aggregates.

**Figure 4:**
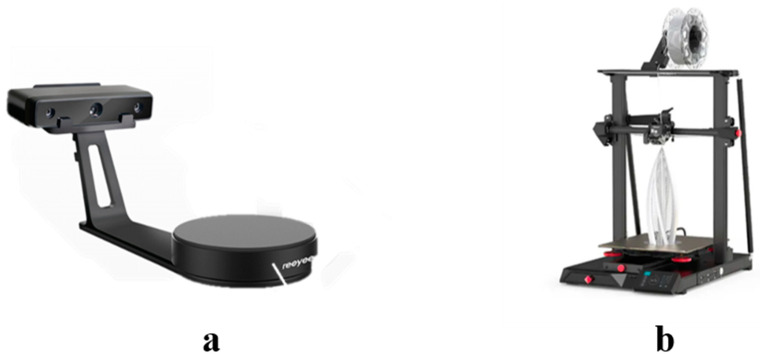
Experimental setup: (**a**). Reeyee Object 3D scanner (**b**). CR-10 Smart Pro 3D printer.

To ensure the accuracy of intelligent aggregate production and data collection, this study employed a 3D scanner to capture various forms of natural aggregates. Suitable shapes were then selected to serve as protective shells (Figure 4a), and 3D printing technology was utilized for their production. Currently, numerous scholars have demonstrated the feasibility of applying 3D printing technology in engineering practice [26,27]. After careful comparison, this study selected the Fused Deposition Modeling (FDM) printer (CR-10 Smart Pro, Shenzhen, China), as illustrated in Figure 4b. Its technical parameters are detailed in Table 6.

**Figure 5:**
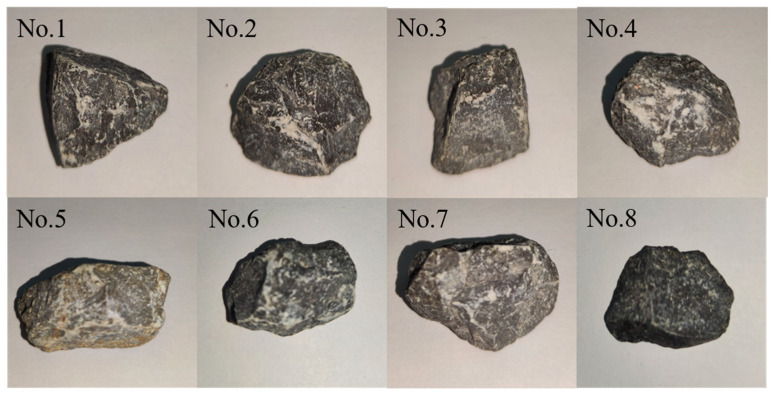
Natural aggregates of different shapes.

Due to the impact of coarse aggregate particle shape on the performance of asphalt mixtures, this study screened various shapes of coarse aggregates during the 3D scanning process, as illustrated in Figure 5. Existing studies on aggregate morphology have demonstrated that needle-shaped and flake-shaped aggregates can significantly affect the performance of asphalt mixtures. Reducing the aspect ratio of aggregates can enhance the high temperature stability of these mixtures [28]. Moreover, the richer the edges of the aggregates, the tighter the bonding will be between the aggregates within the asphalt mixture [29]. Consequently, this article primarily focuses on using convex polyhedron (circles), which effectively simulates the behavior of most real aggregates under mechanical forces. Ultimately, this study utilized natural aggregate no. 2 as the benchmark for modeling and selected polyamide as the material for constructing the packaging shell.

#### 2.1.2. Asphalt and Fillers

Under the same compaction conditions, different asphalt mixtures exhibit varying performance, which can be attributed to the distinct motion responses of aggregates during compaction. Consequently, both the gradation of the mixture and the type of asphalt significantly influence the motion characteristics of the aggregates. To further investigate these motion characteristics throughout the compaction process of asphalt mixtures, this study selected one type of asphalt along with two different gradations.

**Figure 6:**
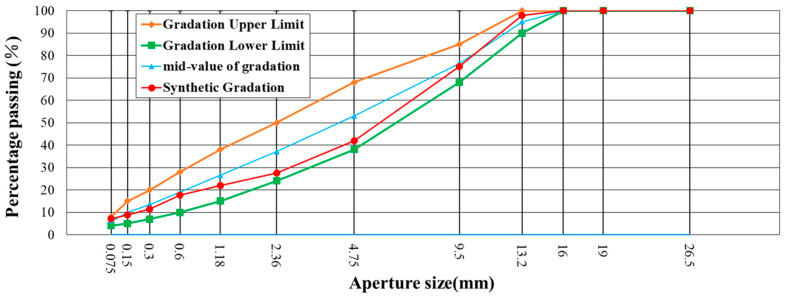
Aggregate gradation of the AC-13 asphalt mixture.

**Figure 7:**
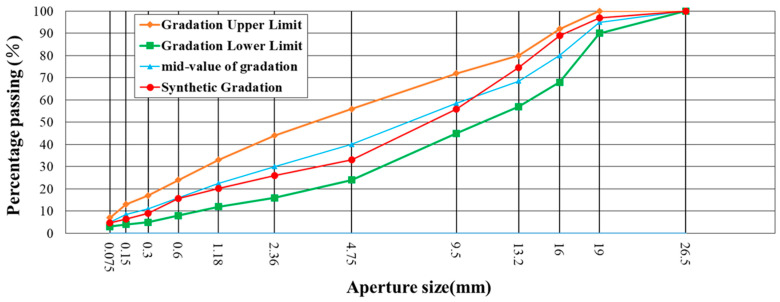
Aggregate gradation of the AC-20 asphalt mixture.

This study utilized two gradations of #70 asphalt, AC-13 and AC-20. AC-13 and AC-20 are typically used as surface layers and intermediate layers in actual road pavement applications. The main difference between them is the maximum aggregate size: AC-13 has a maximum particle size of 16 mm, while AC-20 has a maximum particle size of 19.5 mm. The gradation designs are presented in Table 7 and Table 8. The grading curves are shown in Figure 6 and Figure 7.

### 2.2. Experimental Methods

#### 2.2.1. Intelligent Aggregate Performance Testing

The bearing capacity of asphalt pavement is fundamentally determined by the bonding effect between the asphalt and the aggregates, making excellent interfacial bonding crucial for ensuring pavement performance. The similarity in adhesion between smart aggregates and asphalt, compared to that between conventional aggregates and asphalt, is a key factor that enables intelligent aggregates to effectively simulate the performance of real aggregates in asphalt mixtures. To assess this adhesion, this article employs the traditional water boiling (immersion) method. According to established guidelines, aggregates larger than 13.2 mm should be tested for adhesion using the water boiling method, while aggregates smaller than 13.2 mm should utilize the water immersion method. Consequently, this study applied the water boiling method for adhesion testing.

#### 2.2.2. Compaction Method

**Figure 8:**
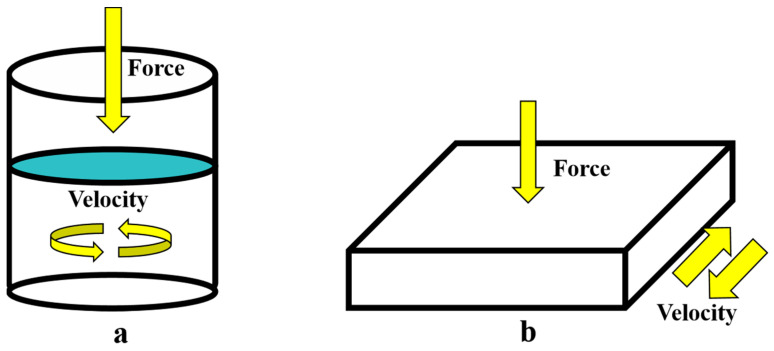
The compaction process of different methods: (**a**) Superpave gyratory compaction; (**b**) wheel rolling method.

In indoor experiments on asphalt mixtures, several compaction methods are commonly employed, including the Marshall method, Superpave gyratory compaction method, wheel rolling method, and vibration method. Among these, the Marshall method is popular due to its simplicity and low cost. However, it does not accurately reflect the compaction process that occurs during actual road construction. The faster forming process associated with the Marshall method can also lead to aggregate breakage, resulting in discrepancies between the test results of Marshall specimens and those of actual road core samples [30]. The Strategic Highway Research Program (SHRP) in the United States found that asphalt mixtures produced using the Superpave gyratory compaction method exhibit the highest correlation with the actual performance of pavement mixtures. In the Superpave gyratory compaction process, in addition to the vertical pressure, the rotation angle of the base creates a cone-shaped formation between the center of the specimen’s surface and the bottom surface, resulting in the generation of horizontal shear forces. The aggregates are rearranged under the influence of vertical pressure and horizontal shear forces to achieve a dense mixture structure. This process also simulates the rolling action of the drum during the actual construction of the mixture [31]. Therefore, the Superpave gyratory compaction method is considered an ideal indoor forming technique for asphalt mixtures. In addition, the wheel rolling method and vibration method are the primary laboratory techniques that closely mimic actual asphalt pavement construction. Dubois et al. [32] examined the void ratios of specimens formed by several compaction methods using gamma rays. The results indicated that the void distribution of core specimens produced by the wheel rolling method was the most uniform, closely resembling the void uniformity of core specimens obtained on-site. This method better represents the compaction process of asphalt mixtures in real pavement engineering. Consequently, this study utilized both the Superpave gyratory compaction method and the wheel rolling method to investigate the motion characteristics of coarse aggregates during the compaction process of asphalt mixtures (Figure 8).

#### 2.2.3. Intelligent Aggregate Layout

**Figure 9:**
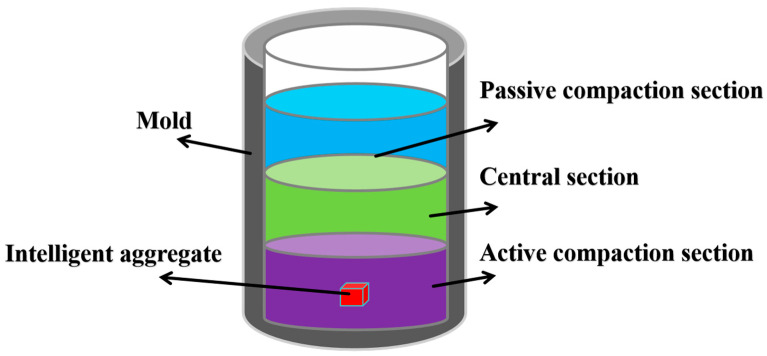
Schematic diagram of intelligent aggregate placement (Superpave gyratory compaction).

**Figure 10:**
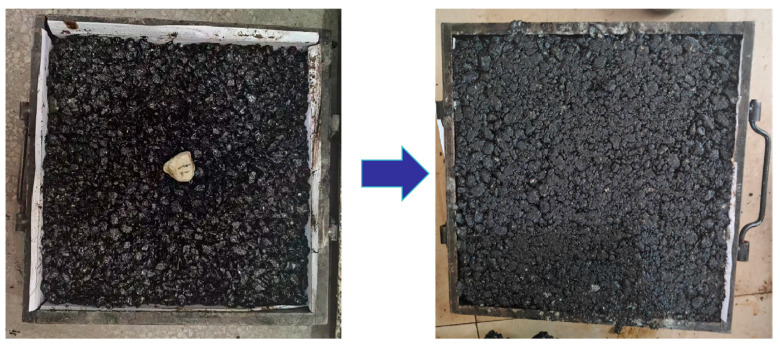
Schematic diagram of intelligent aggregate placement (wheel rolling).

In the course of the compaction process of asphalt mixtures, aggregate particles experience deflection displacement due to external forces, leading to interlocking and squeezing among the particles. This transformation shifts the asphalt mixture from a loose state to a dense state. In laboratory experiments, it is essential to position intelligent aggregates appropriately according to different compaction methods to obtain accurate and efficient data. Over the course of the Superpave gyratory compaction process, the sample height is notably high, and the sample is typically divided into three sections: the active compaction part, the middle section, and the passive compaction part According to Wang et al. [33], the intelligent aggregates in the active compaction area are particularly susceptible to disturbances from external forces, which can lead to their displacement and rotation. In contrast, the aggregates in the passive compaction area are the least likely to be affected by external disturbances. Therefore, in order to investigate the motion characteristics of coarse aggregates during the compaction process of asphalt mixtures, this study selected the active compaction part (center of the lower part of the mold) to place intelligent aggregates (Figure 9). Throughout the wheel compaction process, the rolling wheel moves back and forth. Therefore, in this study, after placing half of the asphalt mixture, the intelligent aggregate was placed at the center of the mold, and then the remaining asphalt mixture was evenly covered on top (Figure 10)

## 3. Results and Discussion

### 3.1. Intelligent Evaluation of Aggregate Adhesion

**Figure 11:**
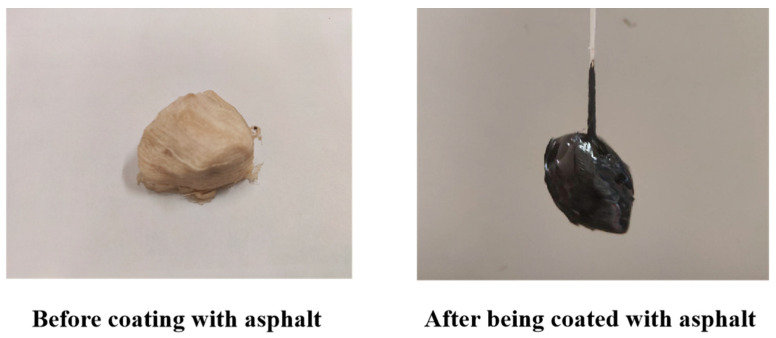
Intelligent aggregate adhesion experiment.

We evaluated the adhesion between asphalt and the smart aggregate encapsulation shell by subjectively comparing the degree of asphalt adhesion on real aggregate (limestone) versus the smart aggregate surface. Also, we observed the extent of asphalt peeling off from the smart aggregate surface, as illustrated in Figure 11.

The adhesion results were obtained through experiments, revealing that both the smart aggregate and the real aggregate exhibited a level 4 adhesion with #70 matrix asphalt. This indicates that the adhesion between the smart aggregate and the asphalt achieved the same performance as that of the real aggregate. Consequently, the experimental accuracy will not be impacted by variations in adhesion.

### 3.2. The Influence of Compaction Methods on Aggregate Movement

In actual road construction, vibratory rollers, static rollers, and rubber-tired rollers are commonly employed. Consequently, the data acquisition frequency was set to 30 Hz. The aggregate was preheated to 160 °C, and the asphalt was heated to 150 °C before being mixed in a mixing pot at 165 °C. To minimize experimental errors, AC-13 gradation was uniformly applied to the asphalt mixtures when investigating the influence of compaction methods on aggregate movement.

**Figure 12:**
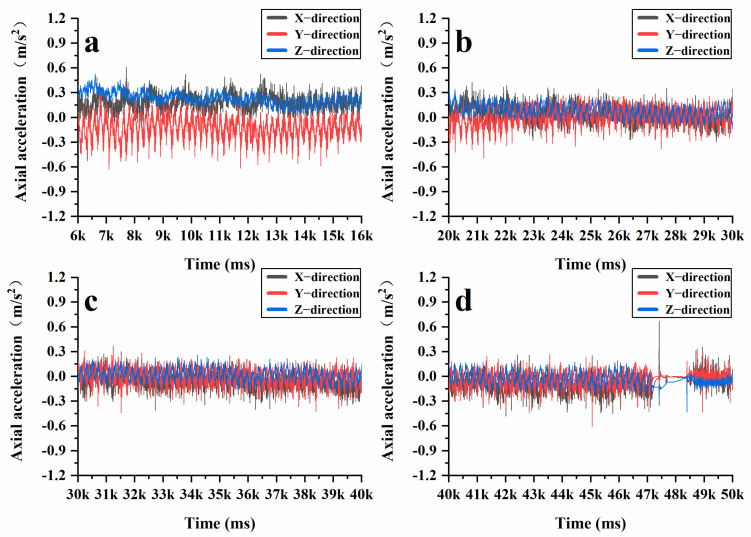
Changes in XYZ direction acceleration at different stages during the rotational compaction process: (**a**) initial compaction stage; (**b**) re-pressing stage; (**c**) re-pressing stage; (**d**) final compression stage.

**Figure 13:**
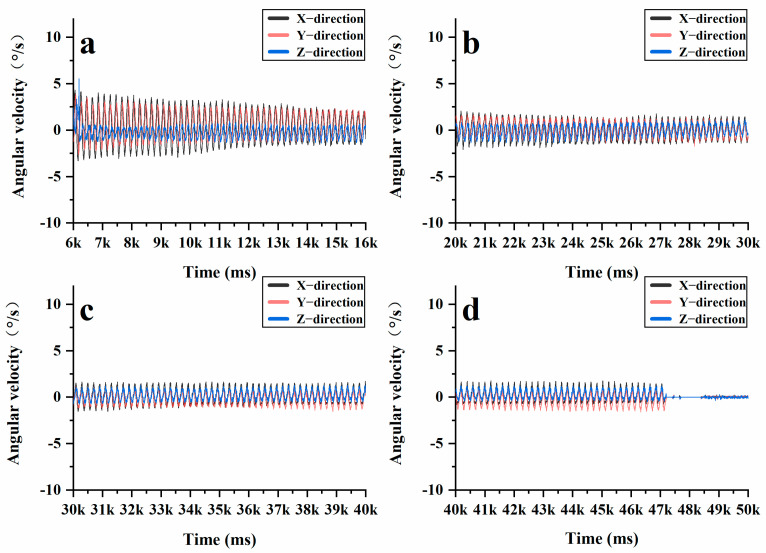
Changes in XYZ direction angular velocity at different stages during the rotational compaction process: (**a**) initial compaction stage; (**b**) re-pressing stage; (**c**) re-pressing stage; (**d**) final compression stage.

In the Superpave gyratory compaction experiment, the rotation angle of the Superpave gyratory compactor was set at 1.16°, the speed at 30 r/min, and the vertical pressure at 600 kPa. The compaction height was established at 150 mm, with a total of 205 compaction cycles and 10 grinding cycles performed. During the Superpave gyratory compaction process, vertical pressure and horizontal shear force work in tandem to compress loose asphalt mixtures into dense specimens. As shown in Figure 12, the acceleration variations of the intelligent aggregates display periodic fluctuations. Figure 12a illustrates the initial compaction stage (0–50 cycles), in which the intelligent aggregates exhibit significant fluctuations in the X, Y, and Z directions. Larger fluctuations are observed in the X and Y directions, indicating that horizontal shear forces are dominant, while there is no significant change in acceleration along the Z direction. However, there is a noticeable change in the height of the asphalt mixture, similar to that observed during static compaction. At this stage, the internal voids within the mixture are large, allowing the coarse aggregates to come into contact and compress one another, thereby forming the primary framework of the mixture. Figure 12b,c illustrate the re-pressing stage (50–200 cycles), at which time the fluctuations of the intelligent aggregates in the X, Y, and Z directions are reduced as the system enters the creep stage. Throughout this phase, a relatively stable framework structure is established among the coarse aggregates, while the fine aggregates and asphalt cement begin to take on the load. This transition marks the stress reaching the interlocking stage. Figure 12d illustrates the final compression stage (after 200 cycles), during which the acceleration fluctuations of the intelligent aggregates in the X, Y, and Z directions are significantly reduced. At this point, the coarse aggregates, asphalt, and fine aggregates bond together to form a stable specimen, leading to a steady state in both density and shear stress. Furthermore, the angular velocity collected by the intelligent aggregates more accurately reflects the three stages that the coarse aggregates undergo during the rotational compaction process. Figure 13a–c illustrate similar trends in acceleration fluctuations. In contrast, Figure 13d demonstrates that throughout the smoothing stage, the angular velocity fluctuations of the intelligent aggregates in the X, Y, and Z directions approach zero. This further confirms that the internal stress of the mixture has reached an interlocking state and that the strength of the specimen has peaked.

**Figure 14:**
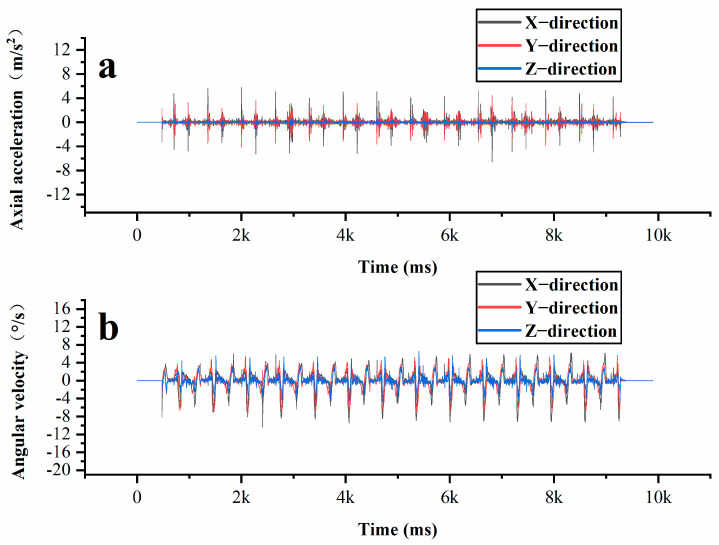
Changes in intelligent aggregate acceleration and angular velocity during the vibration compaction process: (**a**) acceleration variation; (**b**) angular velocity variation.

In the wheel rolling experiment, the rutting board forming instrument was configured for unidirectional rolling, carried out 28 times. The wheel rolling method operates on the principle of vibration compaction. Through the coordinated action of the vibration device and the compaction device, the asphalt mixture is vibrated and compacted within the mold, resulting in a sample with a specific size and shape. In vibration compaction, resonance occurs at specific frequencies and amplitudes of vertical vibration force, leading to significant acceleration and rotation of particles. As shown in Figure 14, the high vibration frequency results in large fluctuations in acceleration, which contrasts with the dynamic response of coarse aggregates during Superpave gyratory compaction. The acceleration and rotation shown by the intelligent aggregates in this context are resonance dynamic responses induced by the vibration force. In the wheel rolling test, manual preloading is necessary after material collection, resulting in a stable contact state among the coarse aggregates throughout the formal compaction process. Consequently, their acceleration and angular velocity display regular, repeated fluctuations.

By comparison, it is evident that compaction methods significantly influence the dynamic response of coarse aggregates in asphalt mixtures. In the case of Superpave gyratory compaction, the horizontal shear exerted by the rotary compactor positively affects the rotation of coarse aggregates, dominating the initial compaction stage. As the coarse aggregates are squeezed against adjacent aggregates, they alter their contact state through self-rotation, leading to considerable fluctuations in acceleration and angular velocity. During the re-compaction and final compaction stages, coarse aggregates establish a stable skeletal structure within asphalt mixtures, making it challenging for them to rotate or change their orientation. As a result, the fluctuations in acceleration and angular velocity of the aggregates exhibit a pattern of regular, repetitive variations. In the wheel rolling method used during testing, the number of wheel rolls is relatively small, the applied stress is high, and manual pre-compaction is performed. This leads to the intelligent aggregates only detecting repeated fluctuations in the regularity of acceleration and angular velocity. Consequently, the Superpave gyratory compaction method is better suited for investigating the motion behavior of coarse aggregates during the compaction process in the laboratory.

### 3.3. Effect of Gradation on the Motion Characteristics of Intelligent Aggregates

During the compaction process of asphalt mixtures, selecting the appropriate mixture type is crucial for ensuring the stability and durability of the pavement structure. Different particle sizes within asphalt mixtures necessitate distinct compaction conditions to achieve the same level of compaction. Therefore, after determining the compaction method, this study focuses on AC-13 and AC-20 asphalt mixtures as the experimental gradation targets to investigate the influence of gradation on the movement characteristics of coarse aggregates.

**Figure 15:**
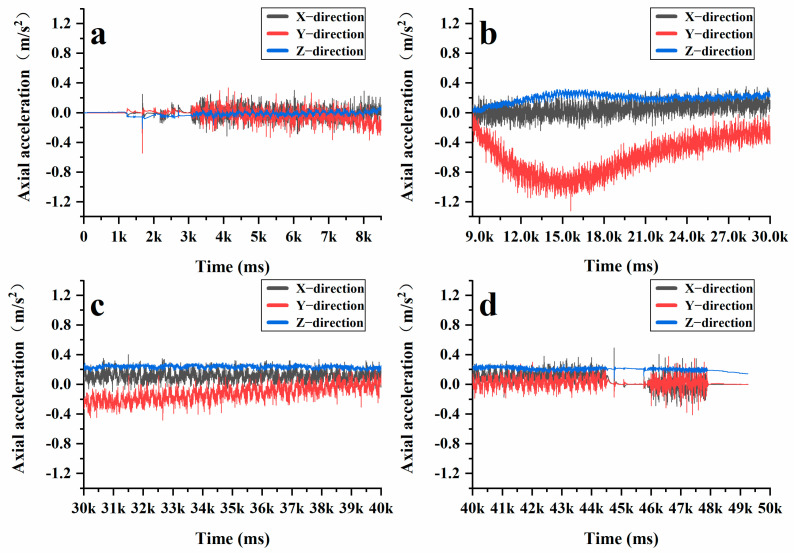
Changes in acceleration of AC-20 intelligent aggregate: (**a**) initial compaction stage; (**b**) re-pressing stage; (**c**) re-pressing stage; (**d**) final compression stage.

**Figure 16:**
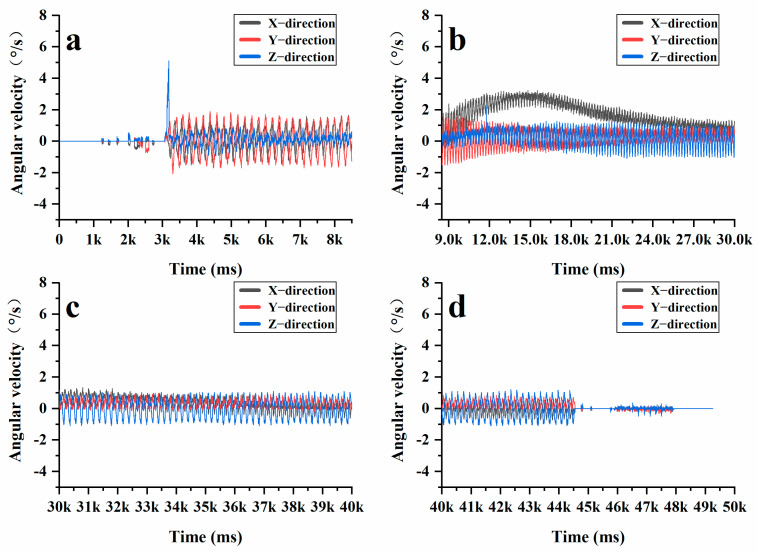
Changes in angular velocity of AC-20 intelligent aggregate: (**a**) initial compaction stage; (**b**) re-pressing stage; (**c**) re-pressing stage; (**d**) final compression stage.

**Figure 17:**
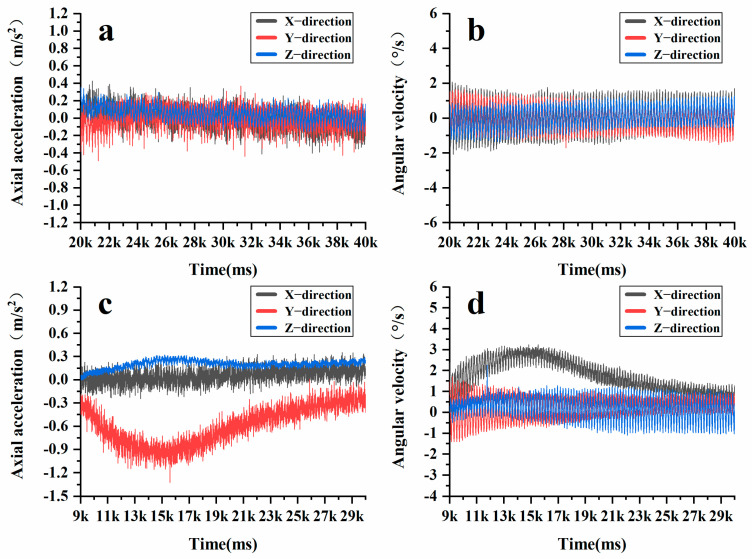
The difference between AC-20 and AC-13 in the re-pressing stage: (**a**) AC-13 acceleration variation; (**b**) AC-13 angular velocity variation; (**c**). AC-20 acceleration variation; (**d**) AC-20 angular velocity variation.

After gaining a clear understanding of the significant vertical displacement of aggregates in the active compaction section of the sample, the intelligent aggregates were positioned within the active compaction section of the AC-20 graded asphalt mixture. Subsequently, the previously mentioned rotary compaction test was repeated under the same compaction conditions. Figure 15 and Figure 16 illustrate the changes in XYZ axial acceleration and angular velocity during the compaction process of AC-20. It is evident that AC-20 experiences significant fluctuations in both acceleration and angular velocity throughout the compaction stage. Figure 17 further compares the XYZ axial acceleration and angular velocity between AC-13 and AC-20 throughout the complex compression stage, revealing pronounced fluctuations in both metrics for AC-20. Under identical conditions, AC-20 contains a higher proportion of coarse aggregates and larger particle sizes compared to AC-13. This results in lower interface lubrication for AC-20, and the larger aggregates contribute to a delayed transition of the asphalt mixture into a dense state. While AC-13 quickly stabilizes as a cohesive skeleton form between its coarse aggregates, AC-20 continues to maintain particle movement. Furthermore, the larger particle sizes present in AC-20 make the coarse aggregates more susceptible to breakage during compaction. Consequently, both the height variation of the mixed sample and the vertical displacement of aggregates in AC-20 are greater than those observed in AC-13.

**Figure 18:**
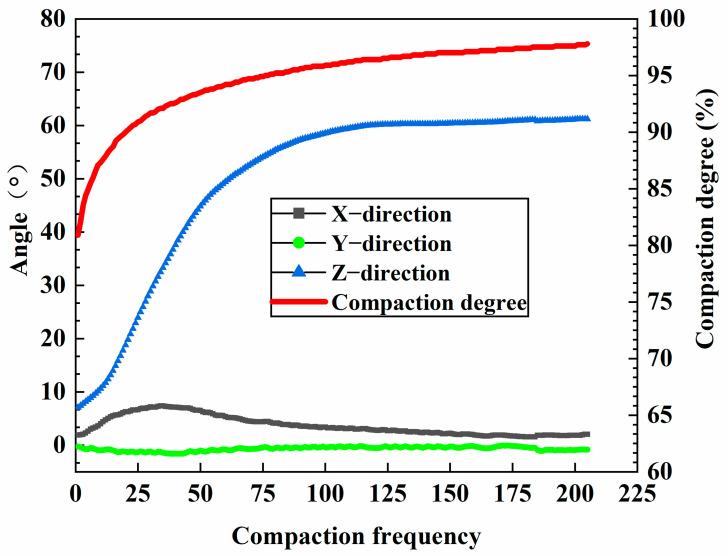
Changes in angle and compaction degree of AC-20 intelligent aggregate.

Additionally, as illustrated by the angle measurements of AC-20 (Figure 18), it is evident that the changes in the XY-axis angles are less pronounced than those in the Z-axis angle during the initial compaction stage. This is primarily due to the large vertical stress encountered throughout this phase, which causes the coarse aggregates to be tightly compressed against one another, thereby forming the structural skeleton of the asphalt mixture. Given the fixed dimensions of the SGC mold, a portion of the vertical stress is converted into lateral stress, resulting in significant fluctuations of the intelligent aggregates in the Z-axis direction. Subsequently, in the later stages of compaction, no significant changes were observed in the XYZ three-axis angles. At this point, the friction between the coarse aggregates increased, thereby limiting their movement. When assessing the compaction degree of an asphalt mixture, a compaction degree of 92% and a porosity of 8% are typically considered the maximum and minimum thresholds for controlling the quality of asphalt pavement compaction. During the mix design, it is generally desirable for the compaction degree to reach 96% or higher, with porosity maintained below 4%. From Figure 17, it is evident that the compaction degree of the asphalt mixture rises rapidly within the first 0–50 cycles, reaching the minimum compaction degree required for quality control of asphalt pavement. Between 50 and 100 cycles, the interlocking and friction forces among the aggregates increase significantly, which restricts the movement of the coarse aggregates and slows down the compaction process. Nonetheless, compaction continues throughout this phase, leading to a further reduction in the height of the specimen and, consequently, an additional increase in the compaction degree. Between 100 and 205 cycles, the internal structure of the asphalt mixture becomes very dense, resulting in only minor changes in height. At this stage, the asphalt mixture reaches a locking point where most of the particles interlock with one another. By examining Figure 15, Figure 16 and Figure 18, it becomes evident that the compaction degree curve aligns closely with the data obtained from intelligent aggregate monitoring. This correlation demonstrates that intelligent aggregates effectively simulate the interactions among coarse aggregates during the asphalt mixture’s compaction process.

Overall, the compaction process of the AC-13 asphalt mixture is more stable, with well-defined boundaries between each stage. The growth in compaction degree and the formation of the skeleton structure in the AC-13 asphalt mixture primarily occur throughout the initial compaction stage. In contrast, the AC-20 asphalt mixture continues to experience a significant increase in compaction degree throughout the compaction process, indicating that the aggregates within the asphalt mixture remain in an active state for the majority of the compaction duration.

## 4. Discussion

In this study, we developed a relatively simple intelligent aggregate to collect motion data of coarse aggregates under different gradations and compaction methods in order to study the motion characteristics of coarse aggregates during the compaction process. However, this intelligent aggregate still has certain shortcomings, as we have not conducted practical applications on pavement and lack application experience. From the perspective of the sensor, we hope to develop a wireless charging sensor designed specifically for asphalt pavements in the future, which could greatly reduce the size of the smart aggregate and extend the lifespan of the sensor.

## 5. Conclusions

In this study, an intelligent aggregate was designed using natural materials to systematically investigate the motion characteristics of coarse aggregates during the compaction process of asphalt mixtures. Two compaction methods and asphalt mixture gradations were employed to compare and analyze the dynamic responses of coarse aggregates. Based on the results and discussions, the following conclusions can be drawn:

1. In the asphalt mixture molding test, the six-axis acceleration sensor proved effective for road monitoring. Additionally, polyamide was identified as a suitable material for intelligent aggregate packaging due to its strong adhesion properties with both asphalt and natural aggregates, such as limestone.
2. The motion characteristics of coarse aggregates in asphalt mixtures are significantly influenced by the selected compaction methods. Impact or vibration generates notable acceleration and angular velocity in the smart aggregates. During the wheel rolling method, resonance dynamic responses occur among the aggregates, leading to continuously varying contact behaviors between the smart aggregates and adjacent ones. In the case of Superpave gyratory compaction, the intelligent aggregates demonstrate distinct fluctuations in acceleration and angular velocity across three stages, which helps to confirm the motion characteristics of coarse aggregates throughout the compaction process of asphalt mixtures.
3. The gradation of asphalt mixtures has a certain influence on the movement characteristics of coarse aggregates during the compaction process. While intelligent aggregates can partially characterize the compaction process of AC-20 gradation, larger particle size aggregates have a greater impact, leading to a longer movement duration of aggregates within the asphalt mixture. This makes it difficult to clearly define the initial compaction, re-compaction, and final compaction stages. In contrast, the movement characteristics of coarse aggregates in AC-13 gradation asphalt mixtures show a distinct regularity during the compaction process.

## Figures and Tables

**Table 1:** Main parameters of the IM600 sensor.

Parameter	Value	Parameter	Value
Module size (mm)	18 × 13 × 2	Weight (g)	1
Temperature range (°C)	−40–125	Acceleration range (m/s^2^)	±16
Use current (mA)	7.6	Angular velocity range (°/s)	±2000
Data collection frequency (Hz)	0.5–250	Angle range (°)	±90
Bluetooth transmission distance (m)	50	Comprehensive range (d)	>20

**Table 2:** Technical parameters of epoxy resin.

Parameter	Value	Parameter	Value
Density (g/cm^3^)	1.1–1.4	Flexural Strength (MPa)	100–120
Compressive Strength (MPa)	107–127	Hot Deformation Temperature (°C)	200

**Table 3:** ABS technical parameters.

Parameter	Value	Parameter	Value
Density (g/cm^3^)	1.06	Printing Temperature (°C)	210–240
Hot Deformation Temperature (°C)	73	Flexural Strength (MPa)	68
Tensile strength (MPa)	40	Bending Modulus (MPa)	1203

**Table 4:** Technical parameters of polylactic acid.

Parameter	Value	Parameter	Value
Density (g/cm^3^)	1.23	Printing Temperature (°C)	170–230
Hot Deformation Temperature (°C)	53	Flexural Strength (MPa)	74
Tensile strength (MPa)	63	Bending modulus (MPa)	1973

**Table 5:** Technical parameters of polyamide.

Parameter	Value	Parameter	Value
Density (g/cm^3^)	1.24	Printing Temperature (°C)	240–260
Hot Deformation Temperature (°C)	120	Flexural Strength (MPa)	122
Tensile strength (MPa)	75	Bending Modulus (MPa)	5160

**Table 6:** Technical parameters of the 3D printer.

Parameter	Value	Parameter	Value
Forming technology	FDM	Printing layer thickness (mm)	0.1–0.35
Print size (mm)	300 × 300 × 400	Hot bed temperature (°C)	≤100 °C
Printing accuracy (mm)	±0.1	Spray nozzle temperature (°C)	≤300
Nozzle diameter (mm)	0.4	Printing consumables	PLA/ABS/PA

**Table 7:** Aggregate gradation of the AC-13 asphalt mixture.

Gradation Type	Percentage by Mass (%) Passing Through Sieve Holes (Square Mesh Sieve/mm)									
	16	13.2	9.5	4.75	2.36	1.18	0.6	0.3	0.15	0.075
Gradation Upper Limit	100	100	85	68	50	38	28	20	15	8
Gradation Lower Limit	100	90	68	38	24	15	10	7	5	4
Production of Synthetic Gradation	100	96.3	76.4	42.8	26.7	16.9	11.3	8.1	6.5	5.9

**Table 8:** Aggregate gradation of the AC-20 asphalt mixture.

Gradation Type	Percentage by Mass (%) Passing Through Sieve Holes (Square Mesh Sieve/mm)											
	26.5	19	16	13.2	9.5	4.75	2.36	1.18	0.6	0.3	0.15	0.075
Gradation Upper Limit	100	100	92	80	72	56	44	33	24	17	13	7
Gradation Lower Limit	100	90	68	57	45	24	16	12	8	5	4	3
Production of Synthetic Gradation	100.0	97.0	89.0	74.5	56.0	33.0	26.0	20.2	15.6	9.1	6.5	4.7

## Data Availability

The raw data supporting the conclusions of this article will be made available by the authors on request. The data are not publicly available due to The overall experiment is not yet completed.
